# Supplementary material for: Oxygen suppression of macroscopic multicellularity
Source: Nat Commun. 2021 May 14;12:2838. doi: 10.1038/s41467-021-23104-0 (PMC8121917; doi:10.1038/s41467-021-23104-0)
Supplement: Supplementary file 1 — Supplementary Information [file 41467_2021_23104_MOESM1_ESM.pdf]

Oxygen suppression of macroscopic multicellularity

G. Ozan Bozdag*, Eric Libby, Rozenn Pineau, Christopher T. Reinhard, and William C. Ratcliff*

*Correspondence to ozan.bozdag@gmail.com or ratcliff@gatech.edu.

Supplementary Information

- **Supplementary Figure 1:** Distribution of cluster size in experimentally evolved snowflake yeast after 145 days of selection for increased size.
- **Supplementary Figure 2:** Confocal images showing ancestral and 145-day evolved snowflake yeast populations for strictly anaerobic treatment group.
- **Supplementary Figure 3:** Confocal images showing ancestral and 145-day evolved snowflake yeast populations for control (mixotrophic) treatment group.
- **Supplementary Figure 4:** Confocal images showing ancestral and 145-day evolved snowflake yeast populations for strictly aerobic treatment group (intermediate O_2).
- **Supplementary Figure 5:** Confocal images showing ancestral and 145-day evolved snowflake yeast populations for strictly aerobic treatment group (supplemental O_2).
- **Supplementary Figure 6.** Survival probability as a function of scaling parameter *k*.
- **Supplementary Figure 7.** Examining model sensitivity to λ_r and *k*.
- **Supplementary Figure 8.** Relationship between oxygen diffusion depth and fittest organism size.
- **Supplementary Figure 9.** Oxygen suppression of multicellular size occurs over a broad range of oxygen diffusion and consumption rates.

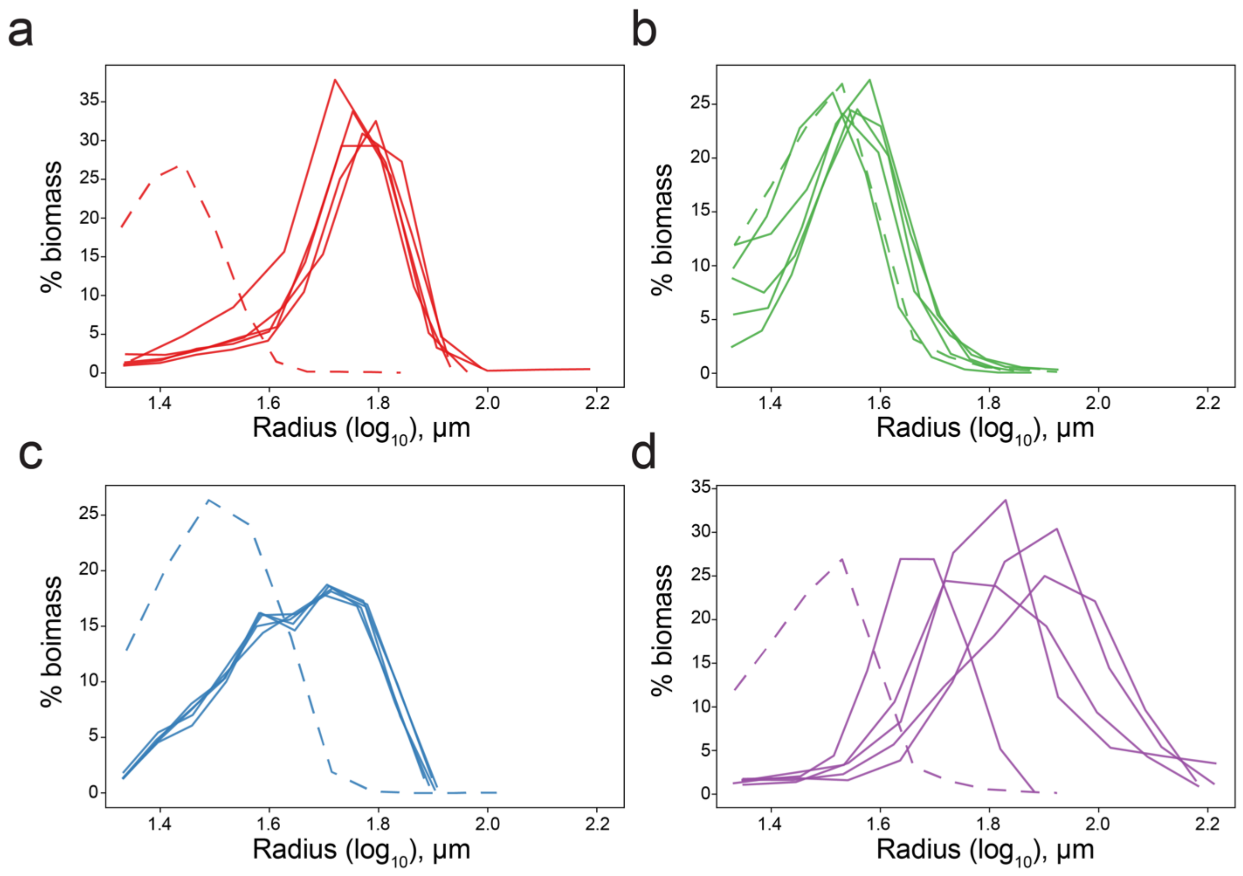

Supplementary Figure 1. Distribution of cluster size in experimentally evolved snowflake yeast after 145 days of selection for increased size (dashed lines show the size distribution of ancestral populations). (**a**) Strictly anaerobic populations, (**b**) strictly aerobic populations, (**c**) control (mixotrophic) populations, and (**d**) strictly aerobic populations cultured under supplemental O_2.

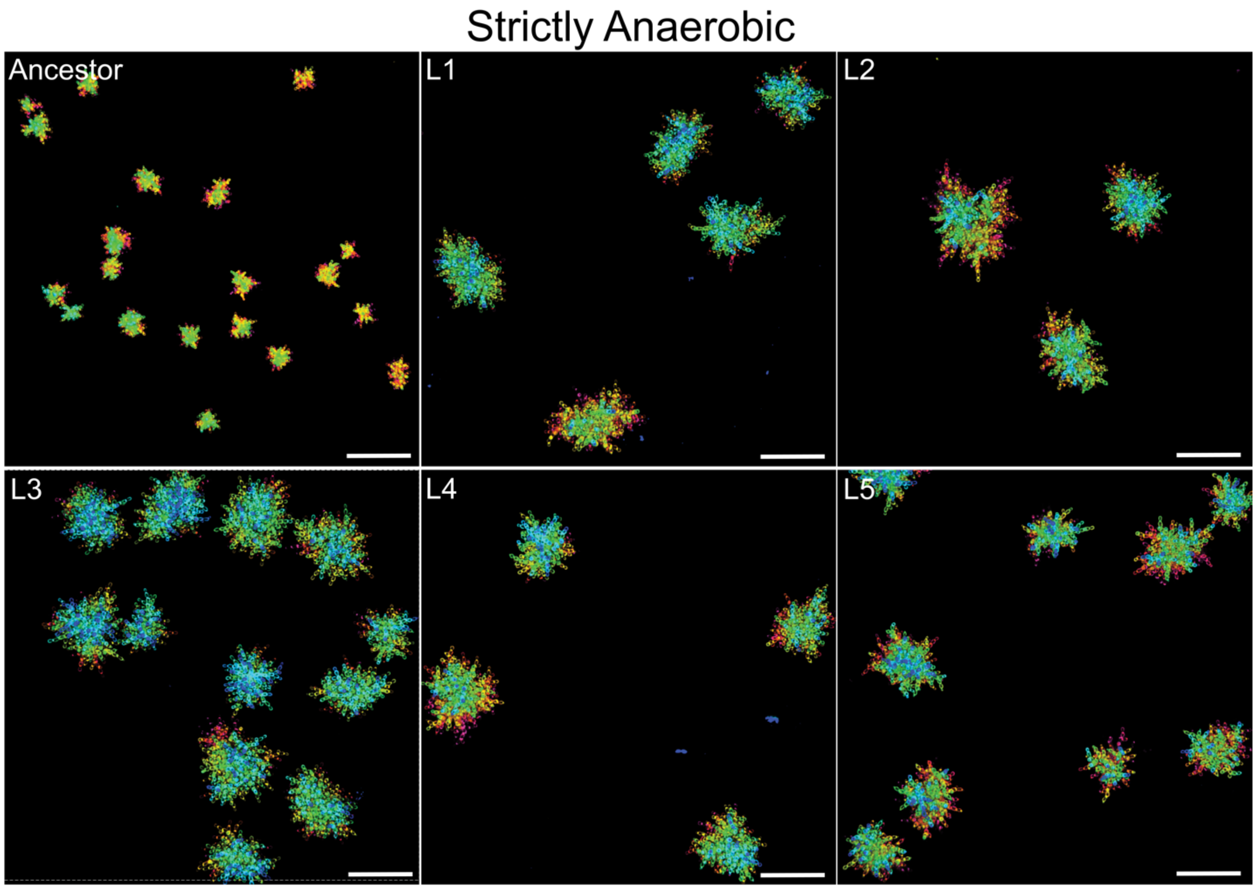

Supplementary Figure 2. Confocal images showing ancestral and 145-day evolved snowflake yeast populations (Lines 1-5) for strictly anaerobic treatment group. Clusters were stained with calcofluor-white as described in Methods. Images were taken at a 100X magnification on a Nikon A1R confocal microscope. Colors represent depth in z-axis. Each frame is 640 x 640 µm, and all scale bars are 100 µm. Images depict a characteristic field of view from each evolving replicate population (n=5).

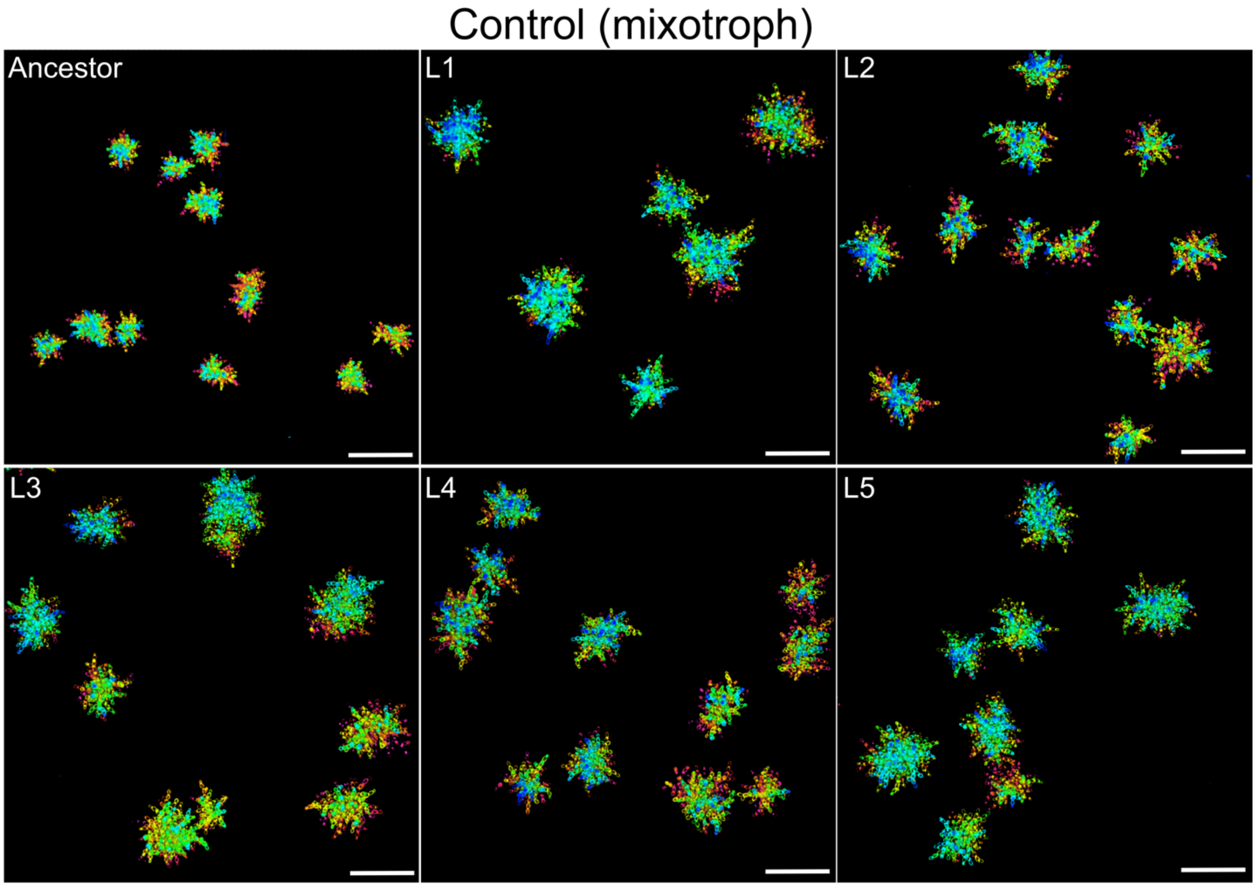

Supplementary Figure 3. Confocal images showing ancestral and 145-day evolved snowflake yeast populations (Lines 1-5) for control (mixotrophic) treatment group. Clusters were stained with calcofluor-white as described in Methods. Images were taken at a 100X magnification on a Nikon A1R confocal microscope. Colors represent depth in z-axis. Each frame is 640 x 640 µm, and all scale bars are 100 µm. Images depict a characteristic field of view from each evolving replicate population (n=5).

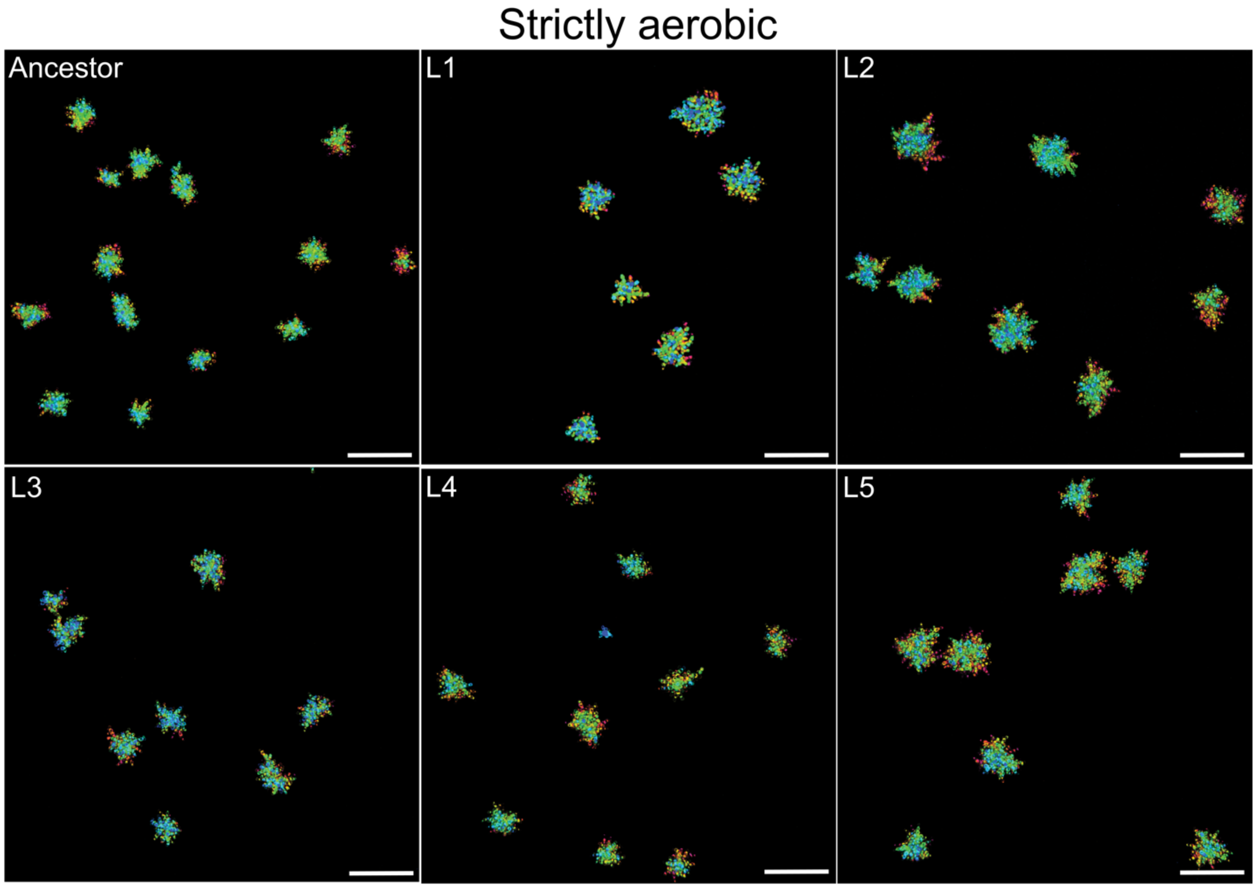

Supplementary Figure 4. Confocal images showing ancestral and 145-day evolved snowflake yeast populations for strictly aerobic treatment group (intermediate O_2). Clusters were stained with calcofluor-white as described in Methods. Images were taken at a 100X magnification on a Nikon A1R confocal microscope. Colors represent depth in z-axis. Each frame is 640 x 640 µm, and all scale bars are 100 µm. Images depict a characteristic field of view from each evolving replicate population (n=5).

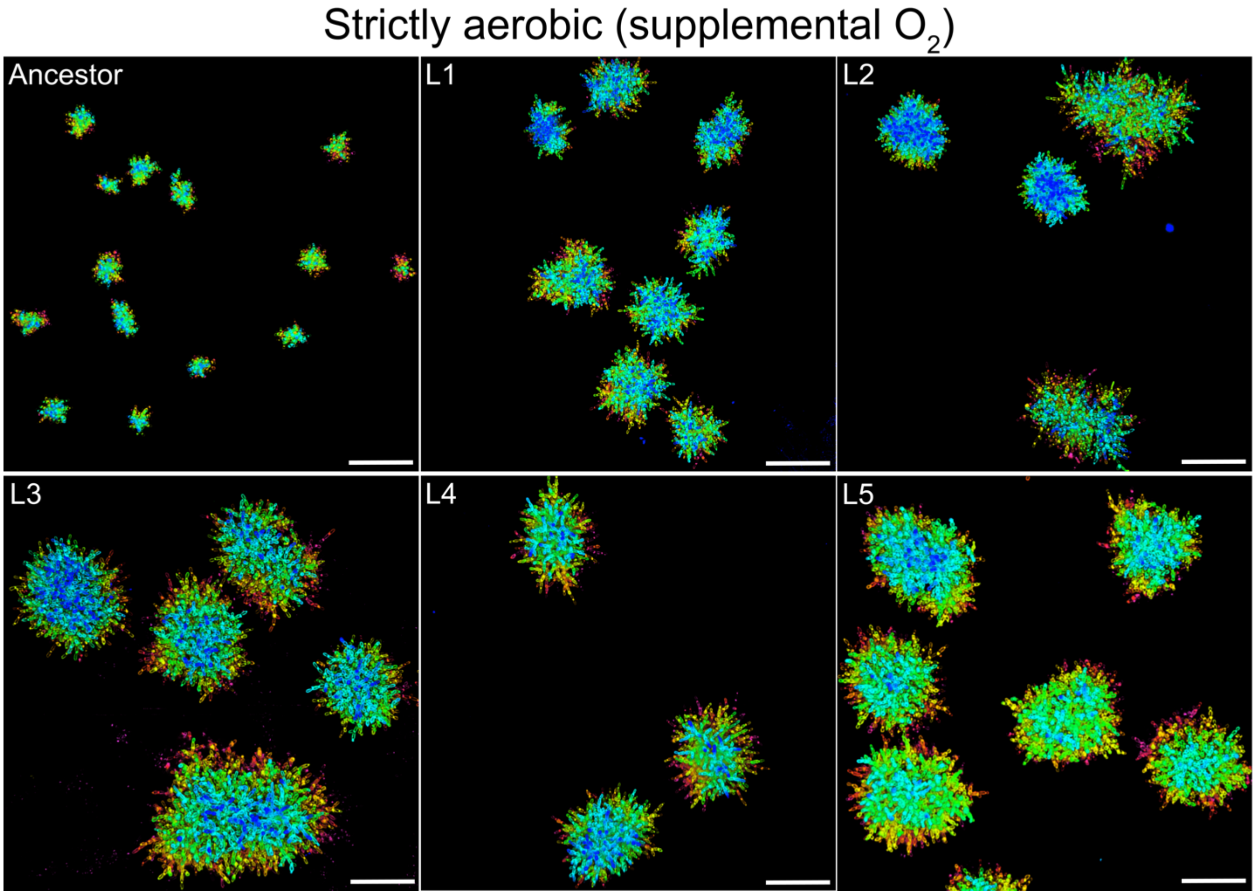

Supplementary Figure 5. Confocal images showing ancestral and 145-day evolved snowflake yeast populations for strictly aerobic treatment group (supplemental O_2). Clusters were stained with calcofluor-white as described in Methods. Images were taken at a 100X magnification on a Nikon A1R confocal microscope. Colors represent depth in z-axis. Each frame is 640 x 640 µm, and all scale bars are 100 µm. Images depict a characteristic field of view from each evolving replicate population (n=5).

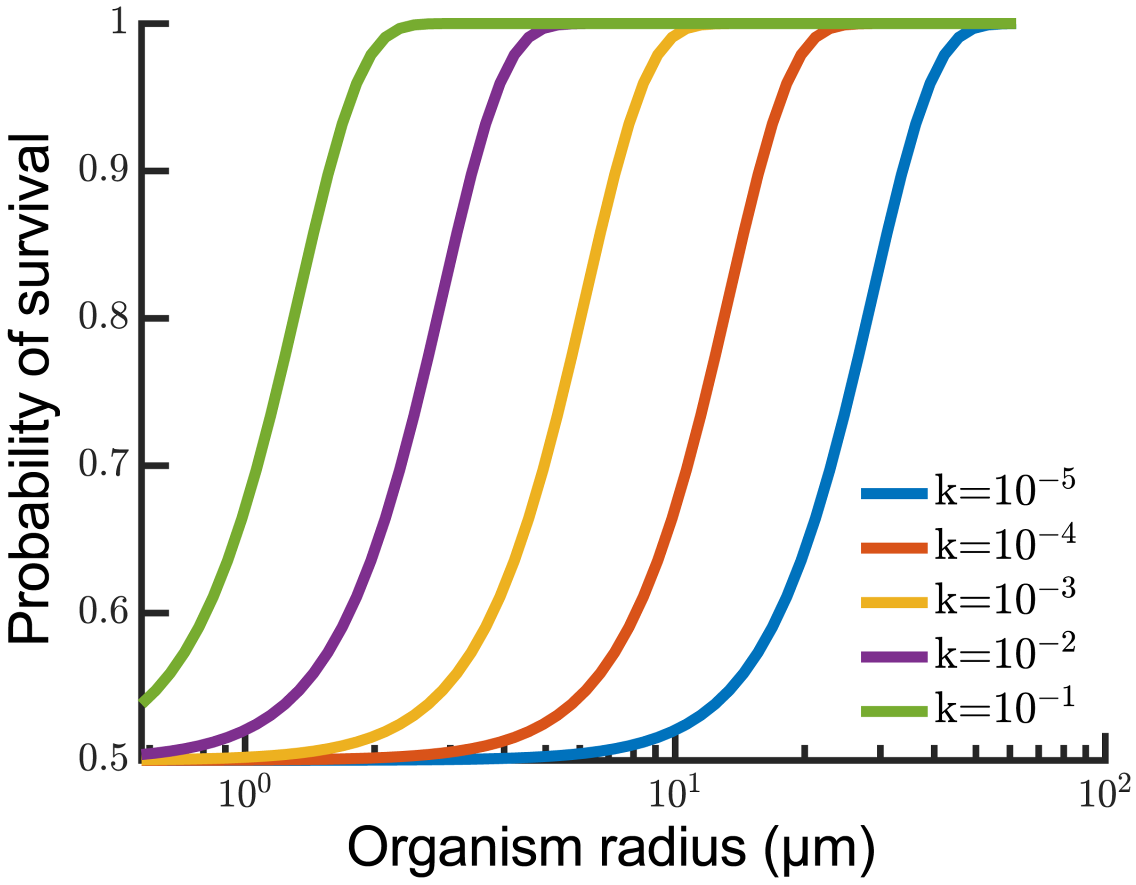

Supplementary Figure 6. Survival probability as a function of scaling parameter *k*. The value of *k* determines the size range over which selection rewards larger organisms. When *k* is large (*e.g.*, 0.1), the selective benefits of increasing size saturate at relatively small sizes. Smaller values of *k* shift the dynamics of size selection towards larger size classes.

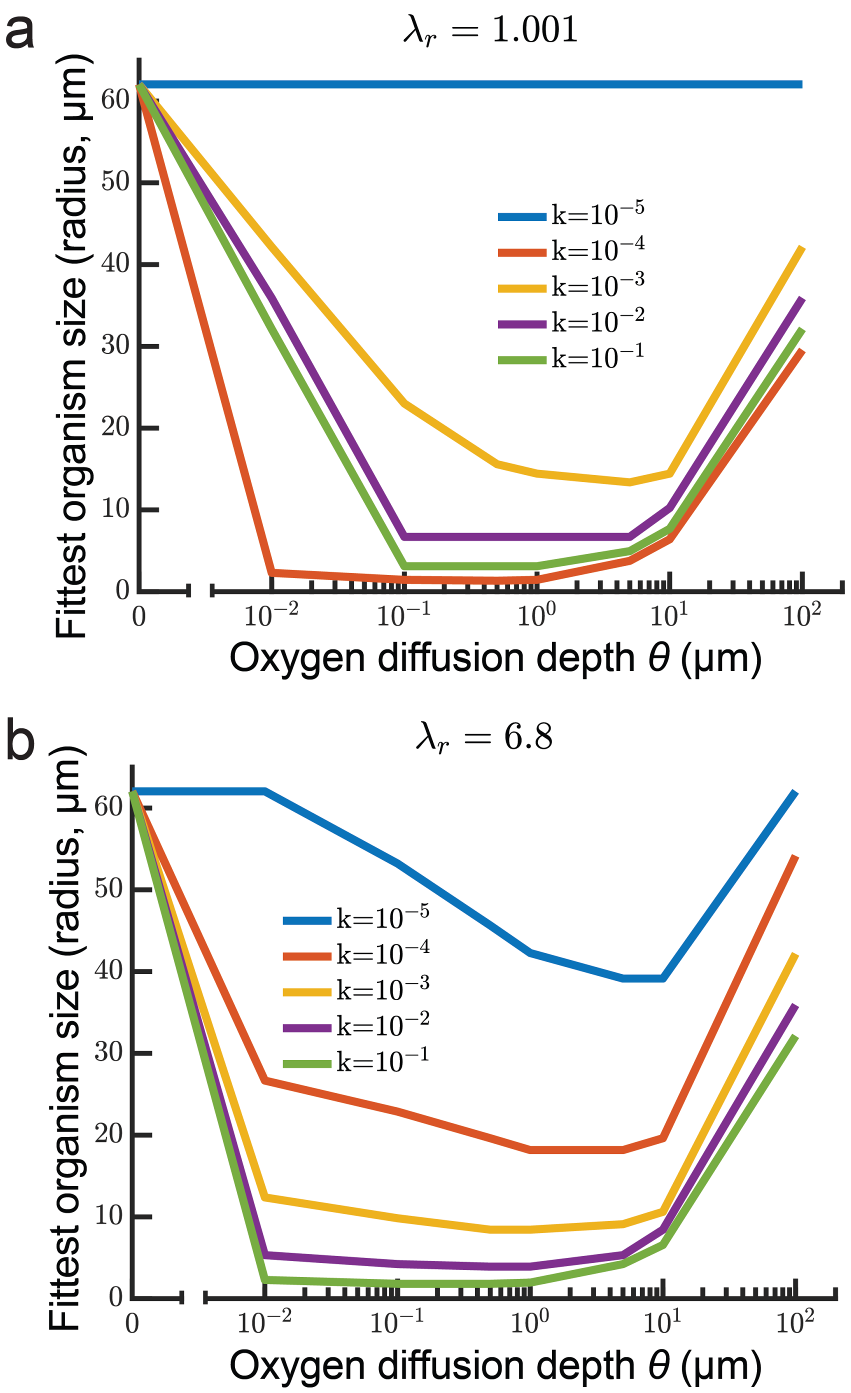

Supplementary Figure 7. Examining model sensitivity to λ_r and k. a) Intermediate oxygen suppresses the evolution of large size even when respiration drives only 0.1% more growth than fermentation (λ_r = 1.001, λ_f = 1). Larger values of k connote that selection acts to reward larger organisms at a smaller absolute size (see Extended Data Figure 6). b) This plot is the same as for (a), but with λ_r = 6.8. The central qualitative result of our model, oxygenic suppression of size, was robust to wide variation in both the benefits of respiration (determined by λ_r) and the size range over which selection acts on to favour larger organisms (determined by k).

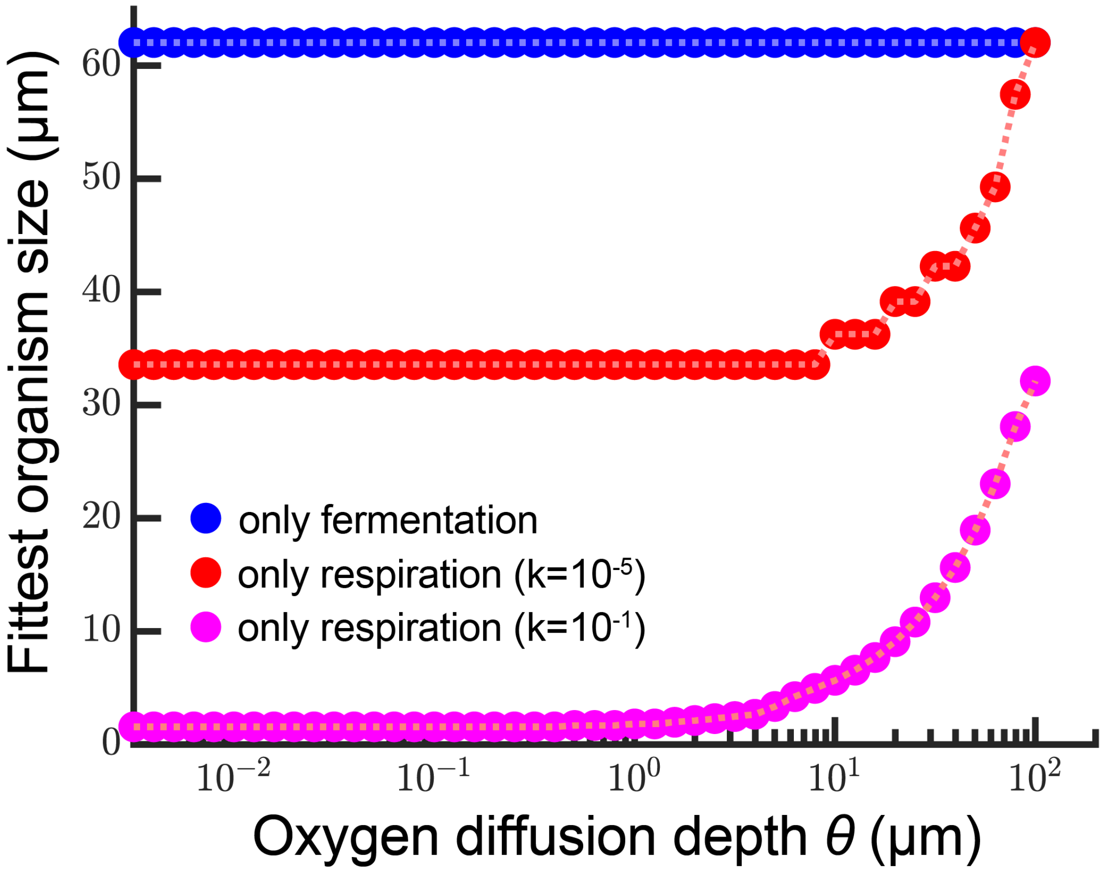

Supplementary Figure 8. In contrast to anaerobic growth, where the maximum size we considered in our model is favored under all O_2 conditions (blue line), large size is only favored in obligate aerobes when oxygen is abundant, allowing oxygen to diffuse more deeply into the organism (θ). The evolution of larger size is more constrained in obligate aerobes than in mixotrophs (see Figure 4c for comparison).

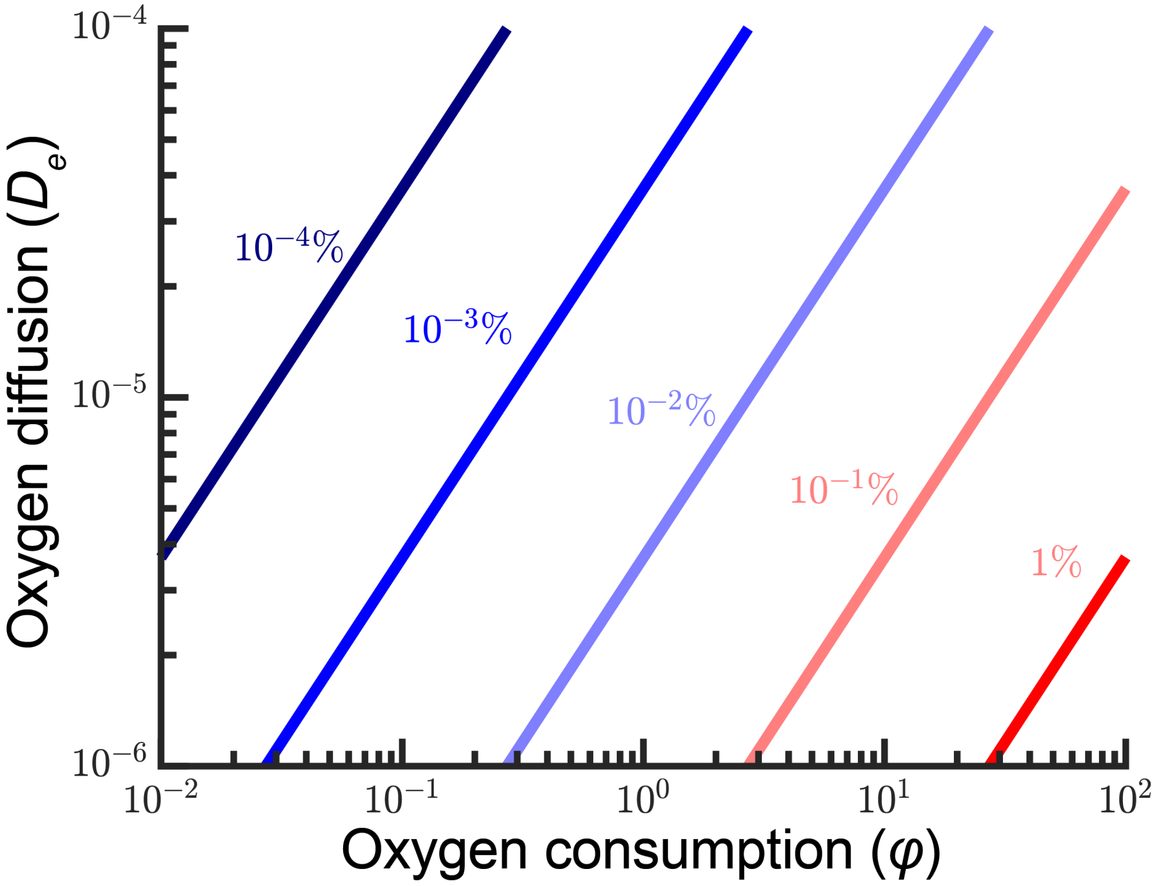

Supplementary Figure 9. Oxygen suppression of multicellular size occurs over a broad range of oxygen diffusion and consumption rates. The above contour plot shows the oxygen concentrations (%pO_2 PAL) that select most strongly for small size (specifically, the oxygen concentration favoring the smallest organismal size for a given value of D_e and φ). Even with 10,000-fold variation in oxygen consumption rates and 100-fold variation in oxygen consumption rates, the transition from an anaerobic to aerobic environment imposes selection for smaller size in our model. Here the k parameter was 0.001 which is in the middle of our range (log-wise).
