# Supplementary material for: Oxygen suppression of macroscopic multicellularity
Source: Nat Commun. 2021 May 14;12:2838. doi: 10.1038/s41467-021-23104-0 (PMC8121917; doi:10.1038/s41467-021-23104-0)
Supplement: Supplementary file 3 — Description of Additional Supplementary Files [file 41467_2021_23104_MOESM3_ESM.pdf]

Description of Additional Supplementary Files

File Name: Supplementary Data 1
Description: List of mutations from a snowflake yeast isolate that had evolved large size.

File Name: Supplementary Data 2
Description: Sequences of all primers used in this study.

File Name: Supplementary Code 1
Description: Matlab code to generate the plots in Figure 4a-4d
