# Supplementary material for: Oxygen suppression of macroscopic multicellularity
Source: Nat Commun. 2021 May 14;12:2838. doi: 10.1038/s41467-021-23104-0 (PMC8121917; doi:10.1038/s41467-021-23104-0)
Supplement: Supplementary file 7 — Reporting Summary [file 41467_2021_23104_MOESM7_ESM.pdf]

nature research

Corresponding author(s): G. Ozan Bozdag & William C. Ratcliff

Last updated by author(s): Apr 9, 2021

Reporting Summary

Nature Research wishes to improve the reproducibility of the work that we publish. This form provides structure for consistency and transparency in reporting. For further information on Nature Research policies, see our Editorial Policies and the Editorial Policy Checklist.

Statistics

For all statistical analyses, confirm that the following items are present in the figure legend, table legend, main text, or Methods section.

n/a	Confirmed	
☐	☒	The exact sample size (*n*) for each experimental group/condition, given as a discrete number and unit of measurement
☐	☒	A statement on whether measurements were taken from distinct samples or whether the same sample was measured repeatedly
☐	☒	The statistical test(s) used AND whether they are one- or two-sided<br>*Only common tests should be described solely by name; describe more complex techniques in the Methods section.*
☒	☐	A description of all covariates tested
☐	☒	A description of any assumptions or corrections, such as tests of normality and adjustment for multiple comparisons
☐	☒	A full description of the statistical parameters including central tendency (e.g. means) or other basic estimates (e.g. regression coefficient) AND variation (e.g. standard deviation) or associated estimates of uncertainty (e.g. confidence intervals)
☐	☒	For null hypothesis testing, the test statistic (e.g. *F*, *t*, *r*) with confidence intervals, effect sizes, degrees of freedom and *P* value noted<br>*Give P values as exact values whenever suitable.*
☒	☐	For Bayesian analysis, information on the choice of priors and Markov chain Monte Carlo settings
☒	☐	For hierarchical and complex designs, identification of the appropriate level for tests and full reporting of outcomes
☒	☐	Estimates of effect sizes (e.g. Cohen's *d*, Pearson's *r*), indicating how they were calculated

Our web collection on statistics for biologists contains articles on many of the points above.

Software and code

Policy information about availability of computer code

Data collection	Cluster size data were collected using Beckman Coulter's (Life Sciences) software (Version: Multisizer software 4). Fitness assay and MitoLoc image data was collected and analyzed using NIS-Elements version 4.30.01 and ImageJ Fiji version 2.1.0 (an open source software). Oxygen concentration data were collected using a FireSting-O2 meter (Pyroscience) and data was recorded by Pyro Data Inspector (version 3.213).
Data analysis	bwa-mem (BWA version 0.1.17), GATK4 HaplotypeCaller (v4.0.3.0), VCFTOOLS (version 0.1.17), SnpEff (v4.3T), Trimmomatic (v0.39), BCFtools-isec (v1.10), and Linux bash shell were used in sequence analysis and data parsing. Prism GraphPad version 8.4.2 for Mac OS was used for statistical analysis. Open source software from SciPy (1.6.0) was used for regression analyses. ImageJ (2.1.0) built-in macro functions were used for image analysis and aerobic activity quantification (Fig 1b). MATLAB (v 9.9) was used to generate and plot results of our mathematical model - we now provide code used to generate plots in Fig4 in Source Code 1.

For manuscripts utilizing custom algorithms or software that are central to the research but not yet described in published literature, software must be made available to editors and reviewers. We strongly encourage code deposition in a community repository (e.g. GitHub). See the Nature Research guidelines for submitting code & software for further information.

Data

Policy information about availability of data

All manuscripts must include a data availability statement. This statement should provide the following information, where applicable:

- Accession codes, unique identifiers, or web links for publicly available datasets
- A list of figures that have associated raw data
- A description of any restrictions on data availability

Source Data are provided with this paper. All strains and microscopy images are available upon request. Whole genome sequencing reads of the large-sized snowflake yeast isolate (i.e., with mutations on ARP5 and GIN4) have been deposited under the BioProject accession number PRJNA719855 at the Sequence Read

Archive (SRA): https://www.ncbi.nlm.nih.gov/sra/PRJNA719855.

Field-specific reporting

Please select the one below that is the best fit for your research. If you are not sure, read the appropriate sections before making your selection.

- [ ] Life sciences
- [ ] Behavioural & social sciences
- [x] Ecological, evolutionary & environmental sciences

For a reference copy of the document with all sections, see nature.com/documents/nr-reporting-summary-flat.pdf

Ecological, evolutionary & environmental sciences study design

All studies must disclose on these points even when the disclosure is negative.

Study description	We evolved snowflake yeast across a range of oxygen levels for larger cluster (multicellular) size. In total, 4 treatment groups with five replicate populations were evolved for ~800 generations or 145 days of selection for size. We then measured each population's cluster size at 0, 50, 100, and 145 time-points. We also measured the daily average oxygen concentration in each replicate by using oxygen optodes. Next, we genetically engineered small and large multicellular yeast and performed a competition experiment between the two phenotypes under low-, intermediate-, and high-oxygen environments and measured the fraction of small vs. large snowflake yeast by imaging populations using fluorescent microscopy. In the second part of the study, we developed a mathematical model to test the generality of our results from the experiments.
Research sample	We used diploid Saccharomyces cerevisiae as a model organism. We deleted a transcription factor, ACE2, in this yeast in order to generate cluster forming phenotype. We used lab strains to facilitate genetic manipulation.
Sampling strategy	We did not employ a specific sampling strategy. A large number of data were collected for each experiment. Each experiment's average sample size is as follows: a) Cluster size data=2000-4000 cluster per population (three replicates per population/time-point), b) aspect ratio data=434 cell per population, c) fitness assay measurements=494 clusters for each replicate were analyzed (number of independent biological replicates was listed in the figure legend), MitoLoc activity measurements = 66 clusters (imaged a total of 66 clusters in 3 different days of their growth cycle).
Data collection	All the data were collected by GOB. Cluster size, aspect ratio, oxygen concentration, and fitness assay data were recorded without any manipulation by the experimenter. Genome sequencing data was generated at the IBB sequencing core facility located at GA-Tech using the Illumina HiSeq-2500 platform. Cluster size data were collected using Beckmen's Multisizer 4e instrument (Figure 2a-c-d). The rest of the multicellular size data were collected by imaging populations of clusters on a Nikon Eclipse Ti inverted microscope (software: NIS-Elements, V 4.30.01) (Fig 3b and 95th percentile size data mentioned in the discussion). We imaged clusters for aspect ratio data collection using a Nikon Eclipse Ti inverted microscope (software: NIS-Elements, V 4.30.01) (Fig 2d x-axis). Relative fitness data were collected by imaging populations of small and large clusters under bright field and red light channels on a Nikon Eclipse Ti inverted microscope (software: NIS-Elements V 4.30.01) (Fig3b). Oxygen concentration was measured for 24 hours for each replicate population and their ancestors using an oxygen meter (FireSting-O2 meter, Pyroscience), and data were recorded by Pyro Data Inspector (version 3.213) (Fig 2c x-axis). Representative images of clusters were taken by using a Nikon A1R confocal microscope (software: NIS-Elements V 4.30.01) (see Fig 2b and Supp. Figures 2-5). All MitoLoc images were captured using a Nikon Eclipse Ti inverted microscope (software: NIS-Elements V 4.30.01) (Fig 1a-b). All data in our mathematical model was generated using MATLAB (V 9.9).
Timing and spatial scale	Data were collected between Jan 2017 - Jan 2021.
Data exclusions	No data were excluded from the analysis. All raw data is shared in Source Data file.
Reproducibility	Cluster size, relative fitness measurements, and aspect ratio analysis were performed on at least three biological replicates. All attempts to replicate these analysis were successful and did not result in the production of outlier values.
Randomization	In order to perform masked sample collection, samples were randomly labeled during all of the data collection stages.
Blinding	Blinding was not relevant to our study because we did not have to make choices based on a phenotypic analysis. We quantified cluster size, aspect ratio values, oxygen concentrations, and relative genotype frequencies from a large sample of populations.

Did the study involve field work? [ ] Yes [x] No

Reporting for specific materials, systems and methods

We require information from authors about some types of materials, experimental systems and methods used in many studies. Here, indicate whether each material, system or method listed is relevant to your study. If you are not sure if a list item applies to your research, read the appropriate section before selecting a response.

Materials & experimental systems

n/a	Involved in the study
☒	☐ Antibodies
☒	☐ Eukaryotic cell lines
☒	☐ Palaeontology and archaeology
☐	☒ Animals and other organisms
☒	☐ Human research participants
☒	☐ Clinical data
☒	☐ Dual use research of concern

Methods

n/a	Involved in the study
☒	☐ ChIP-seq
☒	☐ Flow cytometry
☒	☐ MRI-based neuroimaging

Animals and other organisms

Policy information about studies involving animals; ARRIVE guidelines recommended for reporting animal research

Laboratory animals	Other organisms are used: Saccharomyces cerevisiae - a non-pathogenic microbial eukaryote. Stain: Y55.
Wild animals	n/a
Field-collected samples	n/a
Ethics oversight	n/a

Note that full information on the approval of the study protocol must also be provided in the manuscript.
